# Baseline and Breakthrough Resistance Mutations in HCV Patients Failing DAAs

**DOI:** 10.1038/s41598-017-15987-1

**Published:** 2017-11-22

**Authors:** Stefania Paolucci, Marta Premoli, Stefano Novati, Roberto Gulminetti, Renato Maserati, Giorgio Barbarini, Paolo Sacchi, Antonio Piralla, Davide Sassera, Leone De Marco, Alessia Girello, Mario U. Mondelli, Fausto Baldanti

**Affiliations:** 1 Molecular Virology Unit, Microbiology and Virology Department, Fondazione IRCCS Policlinico San Matteo, Pavia, Italy; 2 Institute of Infectious Diseases, University of Pavia, Pavia, Italy; 3 Division of Infectious and Tropical Diseases, Fondazione IRCCS Policlinico San Matteo, Pavia, Italy; 4 Department of Biology and Biotechnology, University of Pavia, Pavia, Italy; 5 School of Biosciences and Veterinary Medicine, University of Camerino, Camerino, Italy; 6 Division of Infectious Diseases and Immunology, Department of Medical Sciences and Infectious Diseases, Fondazione IRCCS Policlinico San Matteo, Pavia, Italy; 7 Department of Internal Medicine and Therapeutics, University of Pavia, Pavia, Italy; 8 Department of Clinical, Surgical, Diagnostic and Pediatric Sciences, University of Pavia, Pavia, Italy

## Abstract

Sustained virologic response rates have increased dramatically following direct acting antiviral (DAA) therapy in chronic HCV infection. However, resistance-associated substitutions (RASs) may occur either prior to DAA or following drug exposure. The aim of this study was to determine RASs in DAA treatment-failing patients and the role of RASs in failure treatment. Six hundred and twenty HCV patients were evaluated. Direct sequencing of HCV genes was performed at breakthrough in all 31 patients failing DAAs, and in 19 baseline patients. Deep sequencing analysis was performed in 15/19 baseline patients. RASs were detected at breakthrough in 17/31 patients and at baseline in 11/19 patients, although, only 8/19 patients carried RASs associated with the prescribed regimen. Deep sequencing analysis showed RASs at baseline in 10/15 treatment-failing patients. No significant difference was observed with the Sanger sequencing. Treatment failure in the 14/31 patients without RASs was associated with suboptimal treatment. In 54.8% of treatment-failing patients one of the causes of failure might be the presence of RASs. In the majority of patients with RASs, mutations were present at baseline. Direct resistance test is advocated before treatment and at breakthrough in order to optimize retreatment regimens.

## Introduction

Chronic infection with hepatitis C virus (HCV) affects more than 70 million individuals worldwide, with genotype distribution varying according to different geographic areas^1^; these subjects are at risk of developing advanced liver disease and hepatocellular carcinoma^1,2^. After the peginterferon/ribavirin (PegIFN/RBV) era and the introduction of new drugs, chronic HCV infection has become a curable disease. In contrast with other chronic viral infections such as hepatitis B virus (HBV) and human immunodeficiency virus (HIV), the aim of therapy in HCV infection is viral eradication which occurs spontaneously only in a small proportion (20–40%) of patients^3^. Individual variability in immune control of HCV infection explains the huge difference in viral eradication rates^4^. Recently, new antiviral drugs that target specific steps of the HCV lifecycle have been developed. These drugs, termed direct-acting antivirals (DAAs), include NS3/4 A protease inhibitors, NS5B polymerase inhibitors (nucleotide analogues and non-nucleoside inhibitors), and NS5A inhibitors; these are associated with a sustained virologic response (SVR) in ≥90% patients^5–9^.

Although the efficacy of new DAAs is extremely high, the outcome of DAA-based therapies may be negatively impacted by comorbidities or specific HCV characteristics. For example, failure of DAA combinations has been reported to occur more often in HCV patients with advanced cirrhosis, difficult to treat HCV genotypes, high HCV-RNA load, HIV coinfection and an unfavorable IL28B polymorphism^5^. Indeed, accurate genotyping of HCV strains has improved outcomes since most of the current HCV treatment regimens are still strictly genotype dependent^10,11^. Moreover, resistance-associated substitutions (RASs) to DAAs might impair viral response to treatment due to baseline presence and early selection of resistant HCV strains. In fact, HCV generates huge numbers of genetically distinct variants within the seven confirmed genotypes^12^ and mutations, insertions, and deletions responsible for resistance to DAAs accumulate also in the absence of drug selective pressure^13–15^.

The aim of this study was to illustrate potential DAA-resistant variants in HCV NS3, NS5A and NS5B in treated patients at baseline and at breakthrough as well as to underscore the role of RASs in the failure of the firstline DAA treatment or in the re-treatment of patients failing DAAs.

## Results

### Treatment outcome and virologic data

Clinical and virologic characteristics of the patients enrolled in the study are provided in Table 1.

**Table 1:** Patient characteristics by treatment outcome.

	Treatment Outcome	
	Responder patients (%) n° = 589	Treatment-failing patients (%) n° = 31
**Gender**		
Male	386 (65.6)	27 (87)
Female	203 (34.4)	4 (13)
**Race**		
Italian	569 (96.6)	30 (96.7)
Other	20 (3.4)	1 (3.3)
**Genotype**		
1a	91 (15.4)	6 (19.4)
1b	246 (41.8)	9 (29.1)
2	125 (21.2)	4 (12.9)
3	72 (12.2)	8 (25.8)
4	55 (9.4)	3 (9.6)
1b/2k recombinant	0 (0)	1 (3.2)
**No. of HIV-1 co-infected patients**	134 (22.8)	8 (25.8)
**Advanced fibrosis/cirrhosis**	439 (74.6)	27 (87.1)
**Baseline median HCV viral load** (UI/ml log_10_)	5.81 (range 3.07–7.40)	5.78 (range 4.44–6.99)
**IL28B polymorphism**		
CC	92/345 (26.6)	5 (16.1)
CT	196/345 (56.9)	21 (67.8)
TT	57/345 (16.5)	5 (16.1)
Unknown	244	
**Naïve**	246 (41.7)	5 (16.1)
**Peg RBV experienced**	286 (48.5)	22 (71)
**DAA experienced**	58 (9.8)	4 (12.9)

Thirty-one/620 (5%) patients failed to achieve SVR during DAA treatment, 5/31 (16.1%) patients were non responders with detectable HCV RNA for the entire treatment period, while 26/31 (83.9%) patients relapsed during the first month following treatment completion. Of these patients, 5/31 (16.1%) were completely treatment naïve, 27/31(87%) were naïve to DAAs, 4/31 (12.9%) previously received DAA plus peg-IFN/RBV and 22/31 (70.9%) were peg-IFN/RBV experienced (Table 1).

In the comparison of unfavorable (CT or TT) patterns in the IL-28B polymorphism rs12979860, HIV coinfection and cirrhosis no significant difference (P > 0.05) were observed between responders (73.5%; 22.8%; 74.6%) and treatment-failing patients (83.8%; 25.8%; 87.1%) respectively (Table 1).

**Figure 1:**
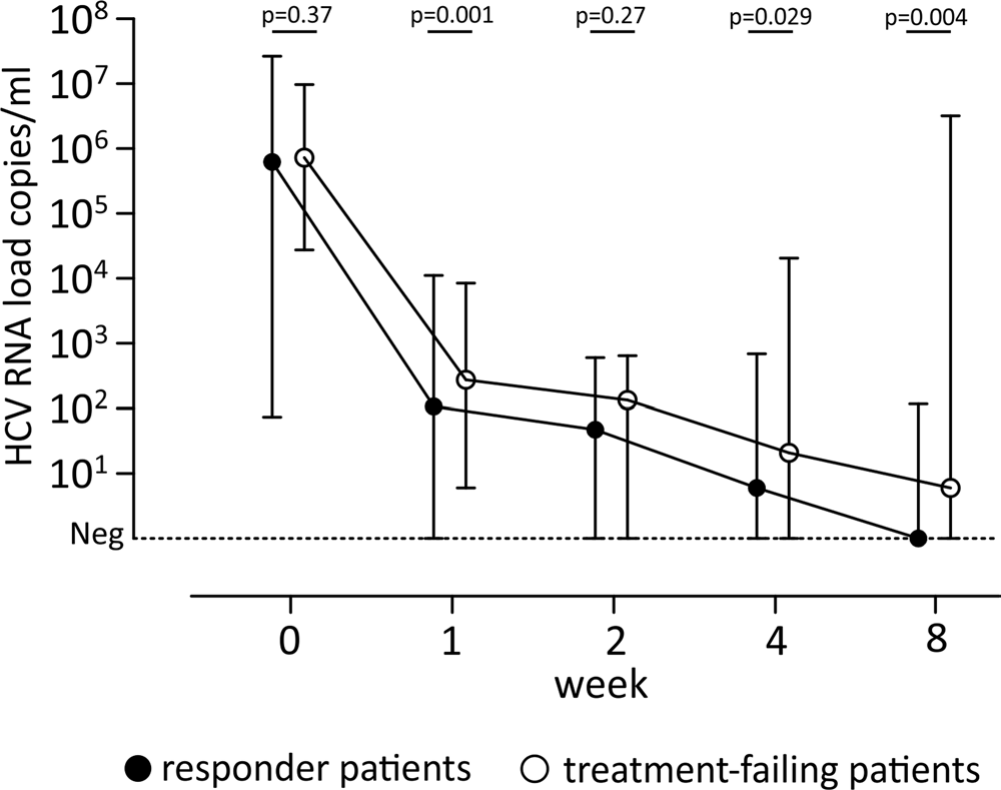
Kinetics of HCV RNA in different group of patients at different time points (week 0, 1, 2, 4, 8). The median HCV RNA level and relative range are reported with black circle for responder and with white circle for treatment-failing patients. Statistic “p” values are reported at each time points.

HCV RNA levels during treatment were significantly different at week 1 (median 107 vs 277, p = 0.001), week 4 (median 6 vs 21, p = 0.02) and week 8 (median 0 vs 6, p = 0.004), between responders and treatment-failing patients, while no difference was observed at baseline (median 6.2 × 10^5^ vs 7.3 × 10^5^, p = 0.37), and at week 2 (median 48 vs 135, p = 0.27) of therapy (Fig. 1). In addition, no difference in HCV RNA levels was observed according to genotype (p > 0.05).

### Sanger-sequencing data analysis

One or more amino acid changes (including RASs and polymorphisms), were observed in 30/31 (96.7%) patients (Table 2). Failure to at least one drug of the ongoing DAA therapy, associated with RASs in at least one of the three target genes, was observed in 17/31 (54.8%) patients (Table 2). Of these, 7/17(41.1%) had RASs to at least two DAA classes. Among the 17 patients failing DAAs 14 (82.3%) were infected with genotype 1a or 1b (Table 2). Sequencing analysis at baseline identified RASs in 11/19 (57.9%) patients (Table 2, in red and green). However, only 8/19 (42.1%) patients (#1, 2, 3, 7, 8, 9, 10 and 13) carried RASs associated with the assigned therapy (Table 2, in red).

**Table 2:** Baseline and post treatment RASs in patients failing DAAs.

**#Patient**	**Genotype**	**Ongoing therapy (weeks)**	**Mutations**			**Drugs with reduced sensitivity**	**Outcome**	**Previous Therapy**
			**NS3**	**NS5A**	**NS5B**			
1 BLPost	1A	Sim+Sof +RBV (12)	**36M**,54S,**155K**,174S **36M**,54S,**155K**,174S	31Mnone	nonenone	Asu,Par,**Sim**,Boc,Tel, Graz,Vel	relapser	Peg/RBV/Telaprevir
2 BLPost	1A	Sim+Sof+RBV (24)	**80K**,174S **80K**,174S	nonenone	nonenone	**Sim**,Tel	Non responder	Peg/RBV
3 BLPost	1A	Omb+Par+Das+RTV+RBV(12)	80K **80K**,**D168V**	**93N93N**	none444D,**556G**	Asu,**Par**,Sim,Graz Dacl,Led,Vel,**Omb**,**Das**	relapser	Peg/RBV
4 BLPost	1A	Omb+Par+Das+RTV+RBV(12)	80K **80K**,**168Y**	none**30R**	none**414T**,444D	**Par**,Dacl,Elb,Led,**Omb**,**Das**,	relapser	Peg/RBV
5 BLPost	1A	Sof+Dacl+RBV(12)	NAnone	NA**30H**	NAnone	**Dacl**,Elb,Led	Non responder	none
6 BLPost	1A	Omb+Par+Das+RTV+RBV(12)	NA**174S**	NA**28V**	NAnone	**Omb**	relapser	Peg/RBV
7 BLPost	1B	Sof+Led(24)	nonenone	none**31I**,**93H**	**159F**,316N **159F**,**282T**,316N,451N	Dacl,Elb,**Led**,Omb,Vel,**Sof**,Das	relapser	Peg/RBV
8 BLPost	1B	Sim+Sof(12)	nonenone	nonenone	**159F**,316N,451S **159F**,316N,451S	Das,**Sof**	relapser	Peg/RBV
9 BLPost	1B	Sim+Sof(12)	36V,**80R**,170V36V,**80R**,**168E**,170V	30R30R	**159F**,316N,451S **159F**,316N,451S,556G	**Sim**,Par,Das,**Sof**	relapser	Peg/RBV
10 BLPost	1B	Sim+Sof(12)	none**168V**	30R,93H30R,31M,93H	**159F**,316N,451S **159F**,**282T**,316N,451S,556G	**Sim**,Par,Led,Dacl,Elb,Vel,Das, **Sof**	Non responder	Peg/RBV
11 BLPost	1B	Sim+Sof(12)	nonenone	30R30R	nonenone	None	relapser	Peg/RBV
12 BLPost	1B	Sof+Dacl(12)	NAnone	NA**93H**	NAnone	**Dacl**,Elb,Led,Omb,Vel	relapser	none
13 BLPost	1B	Sof+Dacl(24)	nonenone	none**31I**,**93H**	**159F**,316N,451Y **159F**,316N,451Y	**Dacl**,Elb,Led,Omb,Vel,**Sof**,Das	relapser	Peg/RBV
14 BLPost	1B	Led+Sof+RBV(12)	NA	NA**31M**,58S,**93H**	NAnone	Dacl,Elb,**Led**,Omb,Vel	relapser	Peg/RBV
15 BLPost	1B	Led+Sof+RBV(12)	none170I	none**31M**,**93H**	none**159F**,316N,451N,556G	GrazDacl,Elb,**Led**,OmbVel,**Sof**,Das,	relapser	Peg/RBV
16 BLPost	1b/2k	Sof+RBV(24)	NAnone	NAnone	NAnone	None	relapser	Peg/RBV
17 BLPost	2C	Sof+RBV(24)	36L,122R,174S36L,122R,174S	30R30R	nonenone	None	relapser	Peg/RBV/Telaprevir
18 BLPost	2C	Sof+RBV(24)	36L,122R,174S36L,122R,174S	30K30R	556G556G	None	relapser	Peg/RBV
19 BLPost	2C	Sof+RBV(24)	NAnone	NAnone	NA553V, 556G	None	Non responder	Peg/RBV
20 BLPost	2C	Sof+RBV(24)	nonenone	30K30K	nonenone	None	relapser	Peg/RBV
21 BLPost	3A	Omb+Par+Das+RTV(12)	36L,168Q36L,80K,168Q	none**93H**	553V,556G553V,556G	Dacl,Led,**Omb**,Elb,Vel	Non responder	Sim+Sof
22 BLPost	3A	Sof+RBV(24)	36L,168Q36L,168Q	93H93H	553V,556G553V,556G	Dacl,Led,Omb,Elb,Vel	relapser	Peg/RBV/Boceprevir
23 BLPost	3A	Sof+RBV(24)	NA36L,168Q	NAnone	NAnone	None	relapser	none
24 BLPost	3A	Sof+RBV(24)	36L,168Q36L,168Q	nonenone	553V,556G553V,556G	None	relapser	Peg/RBV
25 BLPost	3A	Sof+RBV(24)	NA36L,168Q	NAnone	NAnone	None	relapser	none
26 BLPost	3A	Sof+RBV(24)	NA36L,168Q	NAnone	NA553V,556G	None	relapser	Peg/RBV
27 BLPost	3A	Sof+Dacl+RBV(24)	NA36L,168Q	NA**93H**	NA553V, 556G	**Dacl**,Led,Omb,Elb,Vel	relapser	Peg/RBV
28 BLPost	1A3A	Omb+Par+Das+RTV(12)	80Knone	none**93H**	556Gnone	Dacl,Led, **Omb**,Elb,Vel	relapser	Peg/RBV
29 BLPost	4D	Sof+RBV(24)	NA36L, 174S	NA30R,31M	NA414I,553V,556G	None	relapser	Peg/RBV
30 BLPost	4D	Sim+Sof(12)	36L,174S36L,80K,174S	30R,31M30R,31M	414I,553V,556G414I,553V,556G	None	relapser	Peg/RBV
31 BLPost	4D	Sof+pegIFN+RBV(24)	NA36L,174S	NA30R,31M	NA414I,553V,556G	None	relapser	none

RASs to ongoing therapy and drug regimen-including combination with reduced sensitivity are in **Bold**; the RASs not correlated with the ongoing therapy are underlined; polymorphisms are in plain style. BL,baseline. Peg,Pegylated-Interferon; RBV, Ribavirin; RTV, ritonavir; Sim, Simeprevir, Asu, Asunaprevir; Par, Paritaprevir; Graz, Grazoprevir; Dacl, Daclatasvir; Omb, Omitasvir; Elb, Elbasvir; Led, Ledipasvir; Vel, velpatasvir; Das, Dasabuvir; Sof, Sofosbuvir.

In patient #28 different HCV subtypes were detected when comparing HCV strains at baseline and post treatment failure. In 2010 the patient was infected with genotype 3a; subsequently, before starting DAAs in 2015, the viral genotype was determined *de novo* and identified as genotype 1a, thus he was treated on the basis of this genotype. Unfortunately, he was not responsive to treatment and resistance test performed after treatment failure again highlighted the genotype 3a strain carrying variant 93 H. Finally, it must be emphasized that variants in amino acid 282 T were observed in two patients (#7 and 10, Table 2) treated for 12 and 24 weeks, respectively, with treatment including sofosbuvir.

Analysis performed at baseline on the 17 patients carrying HCV variants after treatment showed RASs associated with the DAA regimen in eight (47%) patients. These eight patients carried genotype 1a or 1b strains (Table 2). On the other hand, among the treatment-failing patients (14/31, 45.1%) without RASs at breakthrough, none carried RASs to the ongoing therapy at baseline. The majority of these patients (12/14; 85.7%) infected with genotypes 2, 3, or 4 were treated with a single DAA plus RBV.

### Deep sequencing data analysis

A higher number of HCV variants was observed at baseline with the deep sequencing method (cut-off value, 0.5%) with respect to direct Sanger sequencing (84 vs 57; p = 0.02). All of the 27/84 (32.1%) additional mutations detected with deep sequencing were RASs, and seven of these (25.9%) were associated with the ongoing therapy. RASs observed with the deep sequencing analysis at baseline were detected in 10/15 (66.7%) treatment-failing patients (Table 3); however, no significant difference was observed (p = 0.60) when comparing the number of patients with baseline RASs associated with ongoing therapy, obtained by deep sequencing and Sanger sequencing (11/19, 57.9%). The additional substitutions detected with deep sequencing were observed only in patients infected with HCV genotypes 1a or 1b.. Among these, RASs associated with reduced drug susceptibility detected by deep sequencing (cut-off value, 0.5%) were observed in 5/15 (20%) patients (#3, 4, 8, 9 and 28). Compared with the Sanger sequencing analysis, 2/15 (13.3%) additional patients had RASs associated with the ongoing treatment (#4 carrying 28 T and 556 G mutations and, #28 carrying 43 L and 28 T). Of note, among the five patients with additional RASs associated with reduced drug activity, only the 556 G substitution in patient #3 was selected during treatment. While in patient #10, the additional changes: 31 M in the NS5A gene and 556 G in the NS5B gene were detected after failure by deep sequencing with a frequency of 15.4% and 18% respectively, even in the absence of drug selective pressure. In addition, in patient #1 a mutation in amino acid 31 was detected by both sequencing methods, but was subsequently not selected by the ongoing DAA treatment. Conversely, neither of the variants 168 V in the NS3 gene nor 282 T in the NS5B gene in patient #10, or 31 I and 93 H in the NS5A gene of patient #13, observed post treatment by direct sequencing, were detected at baseline by deep sequencing (Table 3). Instead, when a cut-off value of 1% was applied, in contrast with the 0.5% cut-off value, lower levels of additional RASs were observed at baseline (5/15, 33.3%) in treatment-failing patients, and only 1/15 (6.6%) patient had HCV RASs associated with the ongoing therapy.

**Table 3:** Additional baseline RASs in treatment-failing patients obtained by deep sequencing analysis.

**#Patient**	**Genotype**	**Sanger mutations**			**Additional Deep Sequencing mutations (frequency %)**			
		**NS3**	**NS5A**	**NS5B**	**NS3**	**NS5A**	**NS5B**	**Ongoing Therapy**
**1 BL Post**	1A	**36M**,54S,**155K**,174S **36M**,54S,**155K**,174S	31M none	None none	—	28T(0.5)	556G (0.7)	Sim+Sof
**2 BL Post**	1A	**80K**,174S **80K**,174S	none none	none none	—	28V(5.1),	556G (1.2)	Sim+Sof+RBV
**3 BL Post**	1A	80K **80K**,**D168V**	**93N 93N**	none 444D,**556G**	—	—	556G (0.8)	Omb+Par+**Das**+RTV
**4 BL Post**	1A	80K **80K**,**168Y**	none **30R**	none **414T**, 444D	—	**28T**(0.9),	**556G** (0.5)	**Omb**+Par+**Das**+RTV
**8 BL Post**	1B	none none	none none	159F,316N,451S **159F**, **316N**, **451S**	**122R**(1.8)	93H(1.6)	556G (2.4)	**Sim**+Sof
**9 BL Post**	1B	36V,**80R**,170V 36V,**80R**,**168E**,170V	30R 30R	**159F**,C316N,451S,556G **159F**,C316N,451S,556G	54A(0.7),117H(0.7), **122R**(0.6)	—		**Sim**+Sof
**10 BL Post**	1B	none **168V**	30R,93H 30R,31M93H	**159F**,316N,451S **159F**,**282T**,316N,451S,556G	—	31M(15.4)	556G (18)	Sim+Sof
**11 BL Post**	1B	none none	30R 30R	none none	—	93H(1.3)	556G (0.9)	Sim+Sof
**13 BL Post**	1B	none none	none **31I**,**93H**	**159F**,316N,451Y **159F**,316N, 451Y	122R(7.6),170T(5.8)	—	556G (5.5)	Sof+Dacl
**17 BL Post**	2C	36L,122R,174S 36L,122R,174S	30R 30R	none none	—	—	—	Sof+RBV
**18 BL Post**	2C	36L,122R,174S 36L,122R,174S	30R 30R	556G 556G	—	—	—	Sof+RBV
**21 BL Post**	3A	36L,168Q 36L,80K,168Q	none **93H**	553V,556G 553V,556G	—	—	—	Omb+Par+Das+RTV
**22 BL Post**	3A	36L,168Q 36L,168Q	93H 93H	553V,556G 553V, 556G	—	—	—	Sof+RBV
**28 BL Post**	1A 3A	80K none	none **93H**	556G none	**43L**(0.55),55A(0.8),122R(0.7), 155M(0.5)170T(0.8)174S(0.6)	**28T**(0.5)	—	**Omb**+**Par**+Das+RTV
**30 BL Post**	4D	36L,174S 36L,80K,174S	30R,31M 30R,31M	414I,553V,556G 414I,553V,556G	—	—	—	Sim+Sof

RASs to ongoing therapy and drug regimen-including combination with reduced sensitivity are in **Bold**; the RASs not correlated with the ongoing therapy are underlined; polymorphisms are in plain style. BL,baseline. RBV, Ribavirin; RTV, ritonavir, Sim, Simeprevir, Par, Paritaprevir; Dacl, Daclatasvir; Omb, Omitasvir; Das, Dasabuvir; Sof, Sofosbuvir.

On the other hand, a comparative deep sequencing analysis (cut-off value, 0.5%) performed at baseline in 15 responder patients infected with different HCV genotypes revealed RASs which might be associated with DAAs in 9/15 (60%) patients, although only 4/15 (26.6%) patients (#2, 6, 7 and 13) had HCV RASs associated with the ongoing DAAs (Table 4).

**Table 4:** Baseline RASs in responder patients obtained by deep sequencing analysis.

**#Patient**	**Genotype**	**Deep Sequencing mutations (frequency %)**			**Ongoing Therapy**
		**NS3**	**NS5A**	**NS5B**	
**1 BL**	1A		28V(0.9),31M(6)		Sim+Sof
**2 BL**	1A		28V(0.8),**31M**(1.5)	554S(0.9)	**Led**+Sof+RBV
**3 BL**	1A	170I(98)			Led+Sof+RBV
**4 BL**	1A	80K(98),170I(98)			Omb+Par+Das+RTV+RBV
**5 BL**	1A	170I(98)	28T(0.5),30H(36),93H(24)	556G(0.6)	Sim+Sof+RBV
**6 BL**	1B	**122R**(0.8),174S(99)	30R(96),31I(1.6),93H(20)	556G (1.3)	**Sim**+Sof
**7 BL**	1B	174S(98)	30R(99)	**159F**(99),316N(98),556G(97)	Dacl+**Sof**
**8 BL**	1B	168E(46),174S(99)	30R(99)	556G (6.4)	Led+Sof+RBV
**9 BL**	2C	36L(99),122R(8),170I(100)	28S(0.5),30K(99),30R(0.6),31M(99)	553V(98),556G(99)	Sof+RBV
**10 BL**	2C	36L(99),122R(99),170I(99), 174S(99)	30K(88),30R(11.7),31M(5)	553V(99),554(15),556G(99)	Sof+RBV
**11 BL**	3A	36L(99),168Q(99),170I(98)		553V(99),556G(99)	Dacl+Sof
**12 BL**	3A	36L(99),168Q(99),170I(98)			Dacl+Sof+RBV
**13 BL**	3A	36L(99),168Q(99),170I(98)	28V(12.9),**93H**(5.7)	553V(99),556G(99)	**Dacl**+Sof+RBV
**14 BL**	4D		30R(99),31M(99)	556G(99),561H(98)	Omb+Par+RTV+RBV
**15 BL**	4D	36L(99)			Omb+Par+RTV+RBV

RASs to ongoing therapy and drug regimen-including combination with reduced sensitivity are in **Bold**; the RASs not correlated with the ongoing therapy are underlined; polymorphisms are in plain style. BL,baseline. RBV, Ribavirin; RTV, ritonavir; Sim, Simeprevir, Par, Paritaprevir; Led, ledipasvir; Dacl, Daclatasvir; Omb, Omitasvir; Das, Dasabuvir; Sof, Sofosbuvir.

## Discussion

Patients with chronic hepatitis C are currently treated with a combination of one to three DAAs with or without RBV. HCV DAAs have difference in the resistance barrier. In fact, NS5A, NS3/4 A inhibitors and non-nucleoside inhibitors have relatively low barriers to resistance. In addition, when given as monotherapy, they rapidly select fit resistant variants^16,17^. To increase the chances of successful therapy it is theoretically necessary to accurately evaluate all biological parameters before starting treatment as DAA treatment is clearly genotype dependent. Therefore, correct HCV genotyping and subtyping at baseline should be mandatory^18^. For instance, patient #16 infected with the recombinant form HCV 2k/1b was initially incorrectly assigned to genotype 2, and sofosbuvir/RBV treatment was obviously inadequate for the genotype 2k/1b recombinant which was subsequently correctly identified. Also, coinfection with two different HCV strains is difficult to treat. Similarly, a possible explanation for patient#28’s disease course could be that he was co-infected with both genotypes 1a and 3a and only 1a was initially eradicated despite the presence of RASs at baseline, while genotype 3a persisted since it is notoriously more resistant to current DAA treatment, particularly in patients with advanced fibrosis or cirrhosis^19^.

In this study, factors such as: the favorable IL-28B polymorphism rs12979860, cirrhosis, HIV coinfection and viral load decay during the first two months of therapy had no predictive value for SVR outcome. Although the vast majority of HCV patients responded to therapy, a small subgroup of patients (5%) failed to achieve SVR. In 54.8% of treatment-failing patients (primarily infected with genotype 1a or 1b) one of the causes of failure might be the presence of RASs either at baseline or occurring during treatment as already observed from others^20–22^. Indeed, RASs associated with at least one or two different DAA classes were observed in 54.8% and 41.1% of patients respectively. In contrast, in most of the patients without baseline RASs, treatment failure was probably due to suboptimal efficacy of the single DAA plus RBV. Baseline RASs might be present also in patients with sustained response^17,21,23^. Thus, these mutations *per se* does not appear to hinder a sustained response, however it is possible that multiple factors are involved in relapse patients where such resistance are able to derail therapy especially among patients with HCV genotypes 1.

No correlation between type of outcome (SVR vs failure) and number and type of variants was observed. In fact, the analysis performed at baseline in 4/15 responder patients infected with an HCV strain carrying RASs associated with the ongoing treatment carried a number of variants (including the 93 H mutation) comparable with that observed in treatment-failing patients.

Due to the marked impairment of replicative fitness of HCV strains carrying 282 T variants^24^, relapse has been observed in a very small portion of sofosbuvir-failing patients in many clinical trials^16,20,22^, particularly in patients with repeated and long exposure to the drug^23^. In contrast, patients #7 and #10 carrying HCV variant 282 T were naïve to DAAs and relapsed or did not respond to treatment.

Polymorphisms in DAA naïve patients are more often present at baseline as minor viral populations because they generally reduce fitness^4,16^. Sometimes undetectable of RASs at baseline may be explained by the sensitivity of the direct sequencing technology with an approximate 20% frequency within the HCV quasispecies compared with the 0.5–1% detected by deep sequencing technology. In this study, RASs observed by direct sequencing at the start of treatment in 57.9% of patients could have affected the antiviral therapy outcome. RASs associated with the assigned therapy were detected in only 42.1% of patients. When compared with the Sanger method, 32.1% of additional RASs detected by deep sequencing were observed at baseline among treatment-failing patients. As previously observed with the Sanger method, these substitutions were detected only in patients infected with HCV genotype 1a or 1b probably because they have higher nucleotide variabilities^25^.

When comparing the two sequencing methods, the deep sequencing analysis with its lower (0.5%) or higher (1%) cut-off values detected additional RASs at baseline in 66.7% vs 33.3% of patients, respectively. While additional patients with RASs to the ongoing therapy were observed in 13.3% vs 6.6% of patients, respectively, in agreement with other studies^21,26,27^. It must be emphasized that, except for patient #3, additional mutations detected at baseline by deep sequencing at a very low frequency (<6%) were not subsequently selected for and did not develop during treatment, even when the ongoing drugs could have induced a selective pressure. These findings suggest that, especially when variants are present at low frequencies, they are not always subsequently selected, probably due to reduced replication fitness. In keeping with others^16,21^ the 1% cut off should be considered to avoid an overestimation of RASs. However, it is presently unclear how much clinical relevance should be assigned to given variants for predicting virologic treatment failure^16^. On the other hand, deep sequencing analysis performed at baseline in SVR patients detected RASs in 60% of patients at a frequency between 0.6% to 98%. 26.6% of the patients had HCV RASs associated with the DAA regimen, despite a favorable treatment outcome. Therefore, since RASs detected by deep sequencing analysis were comparable between responder and treatment-failing patients (60% vs 66.7%), and only 13.3% of additional patients would have failed treatment in the group of patients failing DAAs, systematic deep sequencing analysis is currently not recommended^21^. The role of natural resistance is still controversial due to the high SVR rates, and to date it is relevant only for selected NS3-NS5A inhibitors^28–30^, in selected HCV genotypes and clinical settings. Deep analysis on the incidence of RASs at the baseline performed in a larger group of SVR showed that direct sequencing with 15% cut off was able to detect most clinically relevant baseline RASs^21^. On the other hand, based on our data, in which 57.9% of HCV strains at baseline carried RASs in treatment-failing patients, the performance of the direct resistance test at baseline is advocated to detect most clinically meaningful baseline RASs, particularly in patients infected with genotypes 1a or 1b. Recently, some RASs characterized by high fitness persisting as the dominant species for months after treatment cessation have been described^16^. Thus, we believe that evaluation of RASs after treatment failure should be mandatory, in order to optimize retreatment regimens. Despite high SVR rates (95%), the number of future patients with virologic treatment failure will still be significant and most will harbor HCV strains resistant to at least one of the DAA classes. Thus, before switching drugs, resistance data should be included in therapeutic algorithms. However, in the current treatment scenario, majority of DAA treated or retreated patients with baseline resistance will most likely be able to solve the disease^23,31^. In fact, now some of the obsolete DAA combinations employed to treat part of patients in this study would be changed with more efficient formulations including drugs of new generation, able in the majority of them to overcome the problem of baseline RASs.

## Materials and Methods

### Patients

Six hundred and twenty HCV patients with HCV genotypes 1, 2, 3 and 4, referred to our hospital between 2015 and 2016, were included in this study. Characteristics of the patients are described in Table 1. Patients were treated with different combinations of DAA + /-RBV for 12/24weeks (Table 2). A single patient was treated with DAA + pegIFN + RBV (pt#31, Table 2). All patients were compliant to the therapy. Therapeutic decisions were made following the recommendations of the European Association for the Study of the Liver (EASL) clinical practice guidelines during the study period^32^. Serum samples from each patient were prospectively collected at baseline, and at week 1, 2, 4 and 8 during treatment. The study was approved by the Institutional Review Board of the Fondazione IRCCS Policlinico San Matteo (protocol no. 20080009620) and written informed consent was obtained from all study participants. The study was performed according to the guidelines of the Institutional Review Board on the use of biological specimens for scientific purposes in keeping with Italian law (art. 13 D.Lgs 196/2003).

### HCV-RNA quantification and genotyping

HCV genotypes were defined using the Abbott RealTime HCV Genotype II assay (Abbott Park, Illinois, U.S.A.). The NS3/NS5B region was sequenced to further subtype HCV strains when the genotype results were ambiguous. Data were analyzed using the Blast program (http://blast.ncbi.nlm.nih.gov).

Viral load decrease in responder and treatment-failing patients was compared at the baseline, 1, 2, 4 and 8 weeks, and after stopping with the using Abbott HCV-RNA assay (Abbott Park, Illinois, U.S.A.).

Viral RNA was extracted from serum samples using the automatic Easy Mag extractor (Biomerieux, Lyon, France). Full-length HCV NS3/4 A sequences were obtained as previously described [14] while partial HCV NS5A and NS5B genes were amplified using the Transcriptor One-Step RT-PCR enzyme in a nested RT-PCR. Primers used in the RT-PCR and nested PCR, spanning the NS5A and NS5B genes, are available upon request. The PCR products in the first PCR round were obtained by using the outer primers and the following conditions: 30′ at 50 °C for the reverse transcription followed by 10′ at 94 °C, and then 50 cycles at 94 °C for 1′, 55° or 60 °C for 1′ and 68 °C for 2′, with an extension at 68 °C for 10′ in all reactions. Five microliters from the first PCR reaction were used in the nested PCR with the inner primers and the following conditions: denaturation step at 94 °C for 10′ and then 30 cycles at 94 °C for 1′, 60 or 65 °C for 1′ and 72 °C for 2′, with an extension at 72 °C for 10′.

Direct sequencing of PCR products was performed with the Sanger method using an automatic sequencer (ABI PRISM 3100 genetic analyzer DNA Sequencer, Applied Biosystems, Foster City, CA, USA) and the BigDye Terminator v1.1 Cycle Sequencing kit (Applied Biosystems, Foster City, CA, USA). Sequencing primers used to complete the NS5A and NS5B analyses are available upon request. Direct sequencing of the HCV NS3, NS5A and NS5B genes was performed in 31 samples of patients failing DAA treatment and in 19/31 baseline samples.

Nucleotide sequences were assembled using the Sequencer 5.0 (Gene Codes Corp., Ann Arbor, MI) software program and aligned with reference sequences of different subtypes. GenBank accession numbers are as follows: reference sequences AF009606 for HCV genotype 1a, EU155305 for genotype 1b, JX227949 for genotype 2, KU180722 for genotype 3 and FJ462437 for genotype 4.

RASs interpretation with respect to the ongoing therapy was based on the geno2pheno algorithm [http://hcv.geno2pheno.org/index.php] and other clinical and *in vitro* data on RASs interpretation^4,13,16,33^. The sequences reported in this study have been submitted to the GenBank database under accession numbers KY420570-KY420719.

### Illumina MiSeq sample preparation and sequencing

The same PCR products obtained previously for the Sanger sequencing of the NS3/4 A, NS5A and NS5B regions were analyzed using a next generation sequencing (NGS) approach. Samples from 15 DAA failing patients as well as 15 untreated patients with at least 10000 HCV copies/ml were analyzed by NGS.

HCV amplicons (20 µL) were purified using 36 µL Agencourt AMPure XP beads (Beckman Coulter Inc). Amplicon concentrations were determined with the Invitrogen™ Quant-iT™ PicoGreen® dsDNA Assay (Invitrogen, Inc.). Purified amplicons were diluted to 5.0 ng/µl and 2 µL were processed using the Illumina Nextera XT DNA Library Preparation Kit (Illumina Inc.) and uniquely indexed using the Illumina Nextera XT Index Kit (96 Index). All amplified Nextera-PCR products were purified using Agencourt AMPure XP beads (Beckman Coulter Inc.). All recovered DNA was pooled in equal volumes at a normalized concentration (1.43 ng/µl, the lowest concentration recovered). The DNA concentration of the pool was determined using the Invitrogen™ Quant-iT™ PicoGreen® dsDNA Assay (Invitrogen,Inc.). Library quality was determined using a Bioanalyzer 2100 (Agilent Technologies) and was sequenced on a MiSeq deep sequencing platform (Illumina, San Diego, CA) using a the 500*-*cycle MiSeq reagent kit v2.Fastq files were generated using the onboard MiSeq Reporter.

### Deep sequencing data analyses

For the data analysis we collected the DNA sequences of the three analyzed genes (NS3/4 A, NS5A, NS5B,) belonging to the five detected genotypes (1a, 1b, 2, 3, 4). For each sample, we combined the paired end short reads with mothur make.contigs^34^. Combined reads were mapped to the corresponding reference DNA sequence using the Burrows-Wheeler Aligner^35^. The resulting SAM alignment was processed with an in house Python script.

In detail, properly mapped combined reads were selected and located on the corresponding regions of the reference strains. These pairs of DNA sequences were translated into amino acids. Amino acid alignments were analyzed and a table reporting the prevalence of mutations at each reference position was created. Cut-off limits of 0.5% and 1% for prevalence were applied to assess the different impact in clinical evaluation. The presence and prevalence of RASs were also checked, based on the geno2pheno rule set [http://hcv.geno2pheno.org/index.php].

### Bio-containment

All experiments were performed following biocontainment precautions in laboratory with biosafety level 2 (BSL-2).

### Statistical analysis

Continuous variables (i.e. viral load) were compared using the Mann-Whitney U test for independent non-parametric data. Categorical variables were compared by chi-square or Fisher’s exact test, as appropriate. All of the analyses were two tailed and performed using GraphPad Prism version 5 (GraphPad Software Inc., CA, USA); p-values of ≤0.05 were considered statistically significant.
